# P-2094. Difference in Detection Rates of Coagulase-negative Staphylococci between BACTEC^TM^ FX (BD) and BACT/ALERT^®^ VIRTUO^®^ (bioMérieux)

**DOI:** 10.1093/ofid/ofae631.2250

**Published:** 2025-01-29

**Authors:** Jeremy J Taylor, Allison Whalen, Stephanie Colic, Patrick Ozbolt, Stella Antonara

**Affiliations:** OhioHealth Doctors Hospital, Columbus, Ohio; OhioHealth, Columbus, Ohio; OhioHealth Pickerington Memorial Hospital, Pickerington, Ohio; OhioHealth, Columbus, Ohio; OhioHealth, Columbus, Ohio

## Abstract

**Background:**

Blood cultures (BCxs) with skin flora make interpretation of results difficult, impacting patient (pt) care on multiple levels. OhioHealth (OH), a large health care system in central Ohio, transitioned from BACTEC^TM^ FX (BD) to a new BCx detection system, BACT/ALERT^®^ VIRTUO^®^ (bioMérieux) in September 2022 and an increase in coagulase negative staphylococci (CoNS) isolates was noted. We compared detection rates of the most common contaminant, CoNS, between two BCx systems during 3 month pre-and post-implementation periods (March-May 2022 and March-May 2023).

**Methods:**

BCx contamination rates were defined as CoNS in 1/2 sets as per microbiology laboratory standard procedures, or clinically as CoNS in 2/2 sets drawn simultaneously from the same site without signs/symptoms of infection. Pts were excluded if: i) pathogenic organisms were identified in prior BCxs (within 1 week of CoNS) or, ii) any organism other than CoNS was present in the same set. Pure CoNS BCxs were then assessed for number of sets positive (POS) on initial collection and follow up BCxs obtained after the first POS set with CoNS.

**Results:**

From March-May 2022, 23,440 sets were collected with overall positivity rate of 9.5% (2,219 sets). CoNS was identified in 2% of total POS (465 sets). Of those, 244 pts had 1/2 initial sets POS resulting in 28% (69 pts) with redraws. In March-May 2023, 25,314 sets were collected with overall positivity rate of 10.1% (2,564 sets). CoNS was identified in 2.6% of total POS (657 sets). Of those, 364 pts had 1/2 initial sets POS with 37.9% (138 pts) with redraws. Pre-implementation, 55 pts had 2/2 sets POS for CoNS, and 5 pts grew the same organism on redraw. Post-implementation, 78 pts had 2/2 sets POS for CoNS, and 13 pts grew the same organism on redraw. Both periods had similar rates of 2/2 CoNS POS sets drawn simultaneously (72% and 70.5% respectively).

**Conclusion:**

Increased rates of CoNS were detected after transition to the VIRTUO^®^ system, including 1/2 POS sets, resulting in increased follow up cultures. Simultaneously timed sets of BCxs 2/2 POS with CoNS made interpretation of contamination difficult, highlighting the need for education on proper BCx collection techniques, including separate time/site draws to clinically assess true contamination events.

**Disclosures:**

All Authors: No reported disclosures

